# Genome-Wide Characterization of the *ALKBH* Gene Family Reveals a Potential Role of *PgALKBH10* in Multiple Abiotic Stress Responses in *Panax ginseng* C. A. Mey.

**DOI:** 10.3390/genes17070793

**Published:** 2026-07-12

**Authors:** Yiming Sun, Yadong Zhuang, Wanqing Yang, Dan Wang, Jia Hu, Wei Hao

**Affiliations:** College of Medical Technology, Beihua University, Jilin 132013, China

**Keywords:** ginseng, ALKBH, m^6^A RNA methylation, demethylase, abiotic stresses

## Abstract

**Background/Objectives:** N6-methyladenosine (m^6^A) is a prevalent RNA modification that significantly influences various biological processes. AlkB homologs (ALKBHs) belong to the family of specific demethylases and, by regulating m^6^A methylation, are known to be involved in the modulation of plant stress responses. However, the *ALKBH* gene family has not been systematically characterized in ginseng. **Methods:** A genome-wide identification and characterization of the *ALKBH* gene family in ginseng were performed using a telomere-to-telomere reference genome. Phylogenetic relationships, gene structures, conserved motifs, 3D structures, chromosomal distribution, syntenic relationships, *cis*-acting regulatory elements, protein-protein interaction (PPI) networks, and expression profiles were analyzed. Transcriptome datasets covering multiple tissues, developmental stages, cultivars, and abiotic stress treatments were examined. Candidate stress-responsive genes were further validated by qRT-PCR. **Results:** A total of 17 *PgALKBH* genes were identified and classified into seven subfamilies. Structural analyses revealed conserved motifs, exon–intron organization, and 3D structures among members within the same subfamily. Chromosomal localization and synteny analyses suggested that the *PgALKBH* family has been evolutionarily conserved between ginseng and *Arabidopsis* and has primarily undergone purifying selection during its expansion. Promoter analysis identified abundant light-, hormone-, and stress-responsive *cis*-elements. Expression profiling revealed distinct tissue- and developmental stage-specific patterns. The PPI analysis suggested that PgALKBH proteins, especially PgALKBH10, may play a central role in m^6^A-mediated RNA regulation in ginseng. Transcriptome and qRT-PCR analyses further showed that *PgALKBH* genes respond differentially to drought, cold, and salt stresses. Notably, *PgALKBH10* was induced under all three stress conditions. **Conclusions:** This study provides a comprehensive characterization of the *ALKBH* gene family in ginseng and identifies *PgALKBH10* as a promising candidate involved in multiple abiotic stress responses. These findings establish a foundation for elucidating the roles of RNA m^6^A demethylation in ginseng and provide valuable genetic resources for developing stress-tolerant ginseng cultivars.

## 1. Introduction

RNA modifications play a vital role in defining RNA characteristics and functions by influencing its stability, translation, splicing, and transport [1,2]. More than 170 types of RNA modification have been identified [3]. Within the eukaryotic epitranscriptome, N6-methyladenosine (m^6^A) stands out as an exceptionally extensively studied chemical modification of RNA. In RNA molecules, methyltransferases catalyze the methylation of adenosine (A) by substituting the hydrogen atom at the nitrogen-6 (N6) position with a methyl group (CH3) [4]. In plants, m^6^A methylation levels on messenger RNAs (mRNAs) are controlled by three main groups of proteins: readers, writers, and erasers [5]. The writer complex, responsible for m^6^A deposition, consists of methyltransferases including MTA, MTB, FKBP12 interacting protein 37 (FIP37), as well as VIRILIZER and HAKAI [6,7,8,9]. Proteins containing YTH domains, including ECT2-4 and CPSF30-L, act as m^6^A readers by selectively recognizing methylated transcripts and mediating downstream regulatory processes. This specific binding mechanism is fundamental for the control of gene expression and the orchestration of diverse biological processes [10,11]. AlkB homologs (ALKBH) are Fe^2+^ and α-ketoglutarate-dependent dioxygenases that act as specific demethylases. They catalyze the removal of methyl groups from various substrates, including proteins, DNA, and RNA through hydroxylation. The substrate specificity and biological functions of different *ALKBH* family members are influenced by structural variations that affect how they recognize and interact with their targets [12,13].

The *alkB* gene was first identified in *Escherichia coli* in 1983 as a regulator of sensitivity to methyl methane sulfonate [14]. Ever since then, *ALKBHs* have been widely identified across diverse organisms and are increasingly recognized for their roles in RNA epigenetic regulation. In plants, ALKBH-mediated m^6^A demethylation has been implicated in multiple developmental processes. For example, in *Arabidopsis*, *ALKBH10B* regulates m^6^A methylation to control the expression of *FLOWERING LOCUS T (FT)*, *SQUAMOSA PROMOTER BINDING PROTEIN LIKE 3 (SPL3)*, and *SPL9*, thus playing a key role in the floral transition. Plants carrying the non-functional *alkbh10b* allele exhibit a significant delay in flowering onset and impaired vegetative growth [12]. *FvALKBH10B* operates within a complex regulatory cascade involving the abscisic acid (ABA) signaling pathway, where ABA-Responsive Element Binding Factor 3 (FvABF3) induces its expression in strawberries. The mutation of *FvALKBH10B* results in delayed fruit ripening and widespread m^6^A hypermethylation, which impacts numerous genes central to ripening processes. By stabilizing the mRNA of *SEPALLATA3* (*FvSEP3*) through demethylation, *FvALKBH10B* indirectly influences a network of ripening-related genes, including those involved in ABA and anthocyanin biosynthesis [15]. Beyond developmental regulation, ALKBH proteins also play critical roles in plant responses to abiotic stress. The *alkbh10b* mutants of *Arabidopsis* showed increased sensitivity to drought stress, whereas plants overexpressing *ALKBH10B* demonstrated improved drought tolerance. Several m^6^A-modified transcripts associated with the drought stress response exhibited elevated m^6^A levels in the *alkbh10b* mutants. Under dehydration stress, the decay rates of transcripts modified by m^6^A were higher in *alkbh10b* mutants as opposed to the wild type [16]. Similarly, in cotton, *GhALKBH10B* regulates essential drought adaptation networks by lowering the m^6^A levels and mRNA stability of genes related to the ABA and calcium signaling pathways [17]. Moreover, *GhALKBH10* modulates salt stress response genes via the m^6^A demethylation pathway. Silencing *GhALKBH10* leads to elevated expression of salt stress-related genes, including *GhSYTA* (Synaptotagmin A), *GhMDH* (chloroplast malate dehydrogenase), and *GhCIPK6* [18].

Within the pharmacopoeia of traditional Chinese medicine, ginseng is renowned as a botanical of exceptional value and therapeutic importance. The therapeutic value of ginseng is largely attributed to its secondary metabolites, particularly ginsenosides, which exert potent anticancer, anti-inflammatory, and immunomodulatory effects [19,20]. The accumulation of secondary metabolites, as well as overall plant growth and productivity, is strongly influenced by environmental conditions, including abiotic stresses [21,22]. Emerging evidence suggests that epigenetic regulation, particularly m^6^A modification, may play a crucial role in integrating stress signals with metabolic and developmental processes [23,24]. Therefore, elucidating the function of RNA demethylases such as ALKBH proteins in ginseng is of particular importance for understanding the molecular mechanisms underlying both stress adaptation and the formation of medicinal quality traits.

Despite the recognized importance of ALKBH proteins in other plant species, the roles of the *ALKBH* gene family in ginseng remain largely unknown, particularly regarding their involvement in responses to abiotic stress conditions. The recently assembled telomere-to-telomere (T2T) reference genome of ginseng enabled unprecedented assembly continuity and completeness, providing a more reliable genomic framework for the identification and characterization of the *ALKBH* gene family [25]. This research identified 17 potential *PgALKBH* genes within the ginseng genome. We then performed an in-depth characterization, which included their phylogenetic history, genomic localization, exon-intron organization, and promoter-associated *cis*-regulatory elements. In addition, the expression patterns of these *PgALKBH* genes were explored across a range of tissues and organs, as well as in roots at four unique annual growth stages and from 42 cultivated ginseng varieties. Furthermore, the expression of *PgALKBH* genes under different abiotic stresses was verified by Quantitative Real-Time PCR (qRT-PCR). Collectively, this study lays a solid foundation for further investigation into the potential roles of *PgALKBH* genes in abiotic stress adaptation in ginseng.

## 2. Materials and Methods

### 2.1. Identification and Analysis of the Physicochemical Properties of ALKBH Genes in Ginseng

The source data for this work, comprising the T2T reference genome and its gene annotations for a China ginseng accession, were obtained as described in a previous report that documented their public release [25]. The TAIR database (https://www.arabidopsis.org/) served as the primary source for collecting all amino acid sequences corresponding to the *Arabidopsis ALKBH* gene family [26]. Candidate *ALKBH* genes were identified by aligning these protein sequences against the ginseng protein sequences with the blastp algorithm. The 2OG_FeII_Oxy_2 domain (PF13532) within candidate *ALKBH* genes was confirmed via analyzing with the NCBI Conserved Domain Database (CDD) [27]. Further validation of the identified *ALKBH* genes was performed using HMMER [28]. The chromosomal coordinates for each *PgALKBH* gene were retrieved from the annotation files. This locational information was then analyzed with the Toolkit for Biologists integrating various biological data-handling tools (TBtools v2.486) program [29]. The ExPASy proteomics server was employed to determine the fundamental physicochemical properties, specifically the molecular weight (MW) and theoretical isoelectric point (pI) of the ginseng ALKBH proteins [30].

### 2.2. Phylogenetic, Gene Structure, and Conserved Motif Analysis of PgALKBHs

A multiple alignment was produced by aligning the ALKBH protein sequences derived from ginseng, *Arabidopsis*, and wheat. The alignment itself was performed with the MUSCLE algorithm as implemented in MEGA11. The evolutionary relationships were inferred through a phylogenetic tree generated in MEGA11 with the neighbor-joining (NJ) method (Poisson model, 500 bootstraps), which was subsequently rendered and polished using Evolview v2 (https://www.evolgenius.info/evolview-v2/ (accessed on 10 November 2025)). A synteny analysis of the *ALKBH* genes in ginseng was conducted with TBtools v2.486. Furthermore, the MEME program v5.5.8 (https://meme-suite.org/meme/meme_5.5.8/ (accessed on 10 November 2025)) was employed to detect and analyze conserved protein motifs present in the ginseng *ALKBH* family. The TBtools v2.486 platform was also utilized to generate a visual illustration of the architectural features for each gene.

### 2.3. 3D Structure Prediction of PgALKBH Protein

The three-dimensional (3D) structures of the ginseng PgALKBH proteins were predicted using the AlphaFold Protein Structure Database (https://alphafold.com/) [31]. The resulting structural models were then visualized using PyMOL (version 3.1.6).

### 2.4. Chromosomal Distribution and Synteny Analysis of PgALKBHs

The physical positions of the ginseng *ALKBH* genes on the chromosomes were ascertained by referencing the annotation data of the reference genome from Jilin Province, China [25]. TBtools v2.486 was then used to generate a diagram illustrating the distribution of these genes from top to bottom based on their genomic positions. The genome sequence and annotation files of *Arabidopsis* were obtained from the public genomic database Ensembl Plants (https://plants.ensembl.org/index.html (accessed on 10 November 2025)). Bidirectional BLASTP comparisons were conducted using the One Step MCScanX Wrapper in TBtools v2.486 and the resulting synteny file was visualized using the Dual Synteny Plot module in TBtools v2.486.

### 2.5. Identification of Regulatory Motifs in the PgALKBH Gene Promoters

To identify potential *cis*-regulatory elements, the 2000 bp sequences upstream of each *ALKBH* gene’s coding region were first obtained with TBtools v2.486 and defined as the promoters. These promoter sequences were then submitted to the PlantCARE server (https://bioinformatics.psb.ugent.be/webtools/plantcare/html/ (accessed on 10 November 2025)) for motif scanning and prediction [32]. The spatial organization and layout of these elements were afterwards graphically depicted by employing the functionalities of TBtools v2.486.

### 2.6. Protein-Protein Interaction Network Analysis

The protein-protein interaction (PPI) network of PgALKBH proteins and their interacting partners was predicted using TBtools v2.486, based on representative *Arabidopsis* protein sequences. The resulting network was visualized and analyzed in Cytoscape (version 3.10.3) to identify highly interconnected PgALKBH-associated modules within the network [33].

### 2.7. Expression Profiles Analysis of PgALKBHs

Publicly available RNA-Seq datasets (NCBI accession: PRJNA302556), collected from Jilin, China, were utilized for this analysis. The data comprised a wide array of samples, including materials from 14 different ginseng tissues, four distinct age groups (5, 12, 18, and 25 years), and root samples from 42 unique four-year-old cultivars (Table S1). In addition, RNA-Seq datasets associated with abiotic stress treatments were retrieved from the Ginseng Genome Data Resource “http://ginsengdb.snu.ac.kr (accessed on 10 November 2025)”.

Raw sequencing data were first subjected to quality assessment, and adapter sequences as well as low-quality reads were removed using fastp to obtain high-quality clean reads [34]. The reference genome was indexed using HISAT2, and the quality-filtered paired-end clean reads were aligned to the reference genome [35]. The resulting alignments were assembled into transcripts using StringTie, and gene- and transcript-level expression abundances were calculated as transcripts per million (TPM) [36]. In addition, transcript abundance was independently quantified using the pseudo-alignment algorithm implemented in Kallisto to validate the quantification results generated by StringTie [37]. Differentially expressed genes (DEGs) under different stress conditions were identified using DESeq2 [38], with the selection criteria of log_2_FoldChange > 1 and an adjusted *p*-value < 0.01. 

We explored the correlations between the *PgALKBH* genes and cold-, salt-, and drought-stress-related genes in ginseng through correlation analysis. Correlation coefficients were calculated using Origin 2021, and the significance level was judged by the *p*-value (*p* < 0.05).

### 2.8. Plant Materials and Stress Treatments

The Jilin ginseng cultivar “Fuxing No. 2”, sourced from Jilin Province, China, was utilized to generate the adventitious roots used in this study. The plant materials were housed in the Medical Laboratory Testing Technology and Analytical Laboratory of Beihua University.

For drought stress treatments, adventitious roots (cultured for 25 days) were exposed to B5 medium containing 5% PEG 6000. Samples were collected at 0, 3, 6, 9, 12, and 15 days post-treatment. For cold treatment, adventitious roots cultured on B5 medium for 25 days were transferred to 4 °C. Samples were collected at 0, 6, 12, 24, 48, and 72 h post-treatment. For the salt treatment, adventitious roots of uniform size (~1 cm) were placed on B5 medium supplemented with different concentrations of NaCl (0, 70, 80, 90, and 100 mM) and cultured at 25 °C for 30 days. At the conclusion of the experimental treatments, all samples were rapidly frozen using liquid nitrogen and were subsequently held at −80 °C for future gene expression studies.

### 2.9. qRT-PCR Analysis

Total RNA was extracted from the “Fuxing No. 2” variety of Jilin ginseng through the TRIzol protocol. Subsequently, cDNA was synthesized from the purified RNA with the Super RT III Kit provided by Biosharp Biotech (Labgic Tech-nology Co., Ltd., Beijing, China). Relative transcript abundance was then quantified by qRT-PCR, for which all primers were obtained from Sangon Biotech (Shanghai, China) and are listed in Table S2. The *β*-actin gene was utilized as the endogenous reference for all assays. The qRT-PCR reactions themselves were prepared using the SYBR Premix Ex Taq™ II (Tli RNaseH Plus) kit (Takara Biomedical Technology (Beijing) Co., Ltd., Beijing, China). To ensure reliability and reproducibility, qRT-PCR analyses were performed using three independent biological replicates. Relative gene expression levels were calculated using the 2^−ΔΔCT^ method. Statistical analyses and graphical visualizations were performed using GraphPad Prism version 10.1 (GraphPad Software, San Diego, CA, USA). Differences between two groups were evaluated using a two-tailed Student’s *t*-test. Differences were considered statistically significant at *p* < 0.05 and highly significant at *p* < 0.01. Because only pairwise comparisons were performed, no multiple-comparison correction was applied [39].

## 3. Results

### 3.1. Identification and Characterization of ALKBH Genes in Ginseng

A total of 17 *ALKBH* genes were identified in ginseng (Table 1). The molecular features and protein properties of these *ALKBH* genes were analyzed, including gene IDs, number of amino acids (aa), molecular weights (MW), isoelectric points (pI), chromosomal locations, and predicted subcellular localizations. The lengths of the encoded ALKBH proteins ranged from 217 aa (PgALKBH7) to 612 aa (PgALKBH10). Their molecular weights spanned approximately from 24.4 kDa (PgALKBH7) to 67.9 kDa (PgALKBH10). The pI values varied between 4.63 (PgALKBH8B) and 9.51 (PgALKBH2B). Subcellular localization predictions showed that 3 proteins (PgALKBH1D-2, PgALKBH9B-1, and PgALKBH9B-2) were localized in the cytoplasm, and PgALKBH1D-3 was localized in the plastid. Furthermore, a nuclear destination was predicted for the 13 other ALKBH proteins in the set.

### 3.2. Phylogenetic Analysis of ALKBH Genes

**Figure 1:**
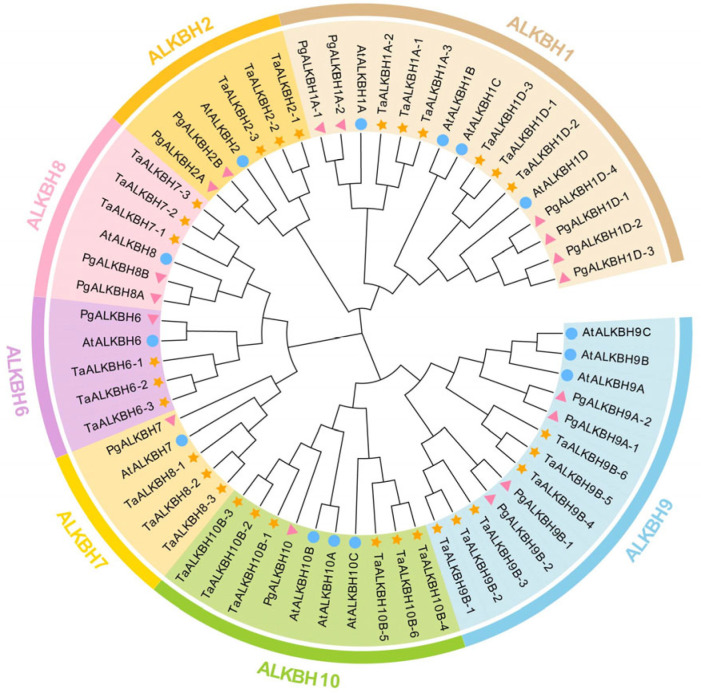
A phylogenetic tree was generated to elucidate the evolutionary relationships of the ALKBH protein family among ginseng, *Arabidopsis*, and wheat. The tree’s construction was based on the NJ algorithm, and statistical confidence for the nodes was assessed via 500 bootstrap iterations. The resulting phylogenetic tree segregated the *ALKBH* genes of ginseng into a total of seven clades. Pg, ginseng; At, *Arabidopsis thaliana*; Ta, wheat.

To elucidate the evolutionary divergence and provide a classification for the *ALKBH* gene family in ginseng, a phylogenetic analysis was undertaken. The tree itself was built based on aligned protein sequences from ginseng alongside those from both *Arabidopsis* and wheat. The *PgALKBH* gene family was divided into seven subfamilies, which are generally categorized according to their homology with *Arabidopsis* ALKBH proteins. Six *PgALKBH* genes (*PgALKBH1A-1*, *PgALKBH1A-2*, *PgALKBH1D-1*, *PgALKBH1D-2*, *PgALKBH1D-3*, and *PgALKBH1D-4*) were classified into the *ALKBH1* subfamily. Four *PgALKBH* genes (*PgALKBH9A-1*, *PgALKBH9A-2*, *PgALKBH9B-1*, and *PgALKBH9B-2*) belonged to the *ALKBH9* subfamily. The *ALKBH2* and *ALKBH8* subfamilies each included two *PgALKBH* genes. *PgALKBH6*, *PgALKBH7*, and *PgALKBH10* were assigned to the *ALKBH6*, *ALKBH7*, and *ALKBH10* subfamilies, respectively. Notably, *PgALKBH10* may serve as a candidate m^6^A RNA demethylase due to its close evolutionary relationship with the well-characterized *AtALKBH10B*, which is known for removing m^6^A modifications from RNA (Figure 1).

### 3.3. Structural and Architectural Characterization of the PgALKBHs

**Figure 2:**
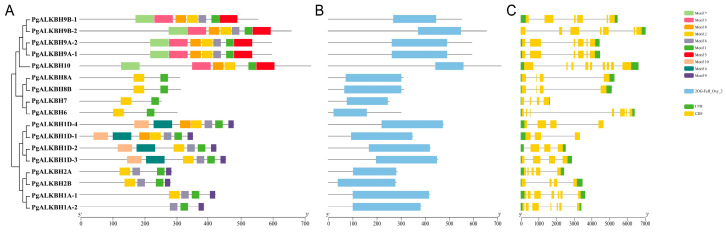
Comprehensive analysis of the ginseng *ALKBH* gene family. This includes an examination of their phylogenetic relationships, conserved protein motifs, domain architecture, and exon-intron organization. (**A**) Evolutionary relationships and conserved motif patterns within the PgALKBH protein family. Each colored box corresponds to a distinct motif. (**B**) Conserved domain of ALKBH proteins in ginseng. (**C**) Gene structures for the *PgALKBH* family. In this schematic, yellow boxes represent the exons, while the intervening introns are indicated by black lines. The untranslated regions (UTRs) are shown as green boxes.

To identify conserved protein motifs, the *ALKBH* sequences were submitted to the MEME online tool. This analysis revealed a total of ten distinct motifs that are broadly shared across the ALKBH protein family. The gene structures were characterized in a separate analysis. All PgALKBH proteins contained motif 1 and motif 2, except PgALKBH1A-2. ALKBH proteins that group together within the same subfamily also exhibit comparable motif compositions, which suggests a degree of functional overlap among these members. Motifs 3, 5, and 7 were unique to the PgALKBH proteins (PgALKBH9A-1, PgALKBH9A-2, PgALKBH9B-1, PgALKBH9B-2, PgALKBH10) assigned to the *ALKBH9* and *ALKBH10* subfamilies (Figure 2A). The presence of the 2OG_FeII_Oxy_2 domain in the PgALKBH proteins was confirmed. In most ALKBH proteins, the 2OG_FeII_Oxy_2 domains are located close to the C-terminal region (Figure 2B). The presence of an identical conserved domain among these PgALKBH proteins suggested potential functional redundancy.

Structural variation was observed across the ginseng *ALKBH* gene family, as determined by an analysis of their exon-intron layouts. The resulting exon count for these genes was found to vary between three and nine. The genes within each subfamily exhibited similar structural characteristics (Figure 2C). A notable structural intricacy and variety was observed among the candidate genes across the various subfamilies. This finding implies that the *PgALKBH* genes in ginseng have probably retained their primary functions but could have also evolved to perform new biological tasks.

### 3.4. 3D Structure Analysis of OsALKBH Proteins

**Figure 3:**
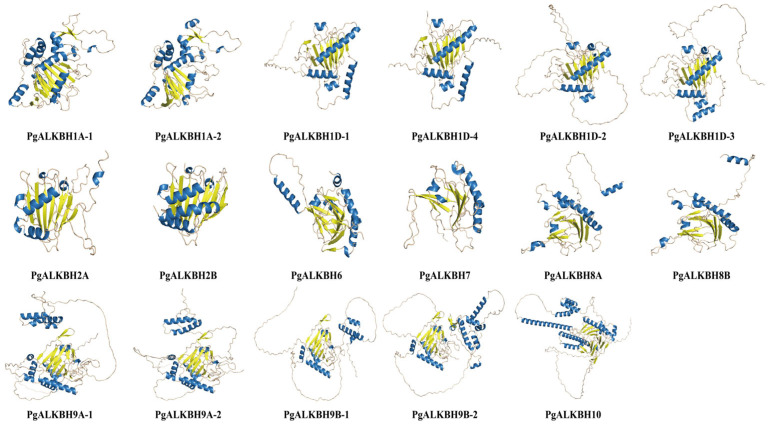
3D structural models of PgALKBH proteins. α-helices, β-sheets, and random coils are represented by blue helices, yellow arrows, and wheat lines, respectively.

To further investigate the structural characteristics of the PgALKBH proteins, their 3D structures were predicted using AlphaFold (Figure 3). All 17 PgALKBH proteins exhibited well-defined tertiary structures composed predominantly of *α*-helices and β-sheets. Proteins belonging to the same subfamily displayed highly similar overall folding patterns. For example, PgALKBH1A-1 and PgALKBH1A-2 exhibited nearly identical structural architectures, as did PgALKBH9A-1 and PgALKBH9A-2, as well as PgALKBH9B-1 and PgALKBH9B-2, indicating a high degree of structural conservation following gene duplication. Structural variations were observed among different PgALKBH subfamilies, suggesting potential functional divergence among these proteins. Despite sharing conserved domain architectures, the distinct structural features may contribute to their diverse biological functions.

### 3.5. Genomic Localization and Synteny Analysis of the PgALKBHs

**Figure 4:**
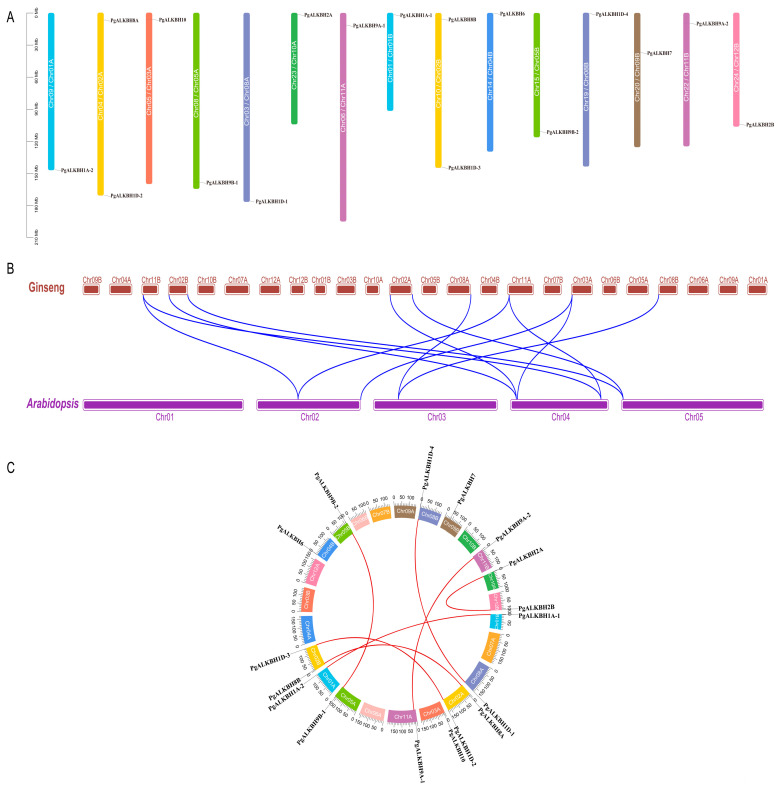
Genomic distribution and synteny analysis of the ginseng *ALKBH* gene family. (**A**) Chromosomal localization of *PgALKBHs* across both subgenomes A and B. (**B**) Synteny analysis of *ALKBH* genes between ginseng and *Arabidopsis*. The blue lines indicate syntenic *ALKBH* gene pairs. (**C**) Collinearity analysis of *PgALKBHs* in ginseng chromosomes. The red lines indicate collinear pairs of *PgALKBH* family genes.

Ginseng is known to be an allotetraploid, and earlier studies consequently partitioned its chromosome set into subgenome A and subgenome B. The physical positions of the ginseng *ALKBH* genes within the genome were ascertained by conducting a search with the BLASTN program. The *PgALKBH* genes were approximately evenly distributed between the two subgenomes, with 8 genes anchored on subgenome A and 9 genes on subgenome B. Chromosomes 4 and 10 each harbor two *PgALKBH* genes, while chromosomes 1, 3, 5, 6, 8, 9, 14, 15, 19, 20, 22, 23, and 24 each contain a single *PgALKBH* gene. Eight *PgALKBH* genes are located on corresponding chromosomes across subgenomes A and B (Figure 4A). All *PgALKBH* genes are positioned near the terminal regions of the chromosomes, except for *PgALKBH7*. These results indicated that ginseng’s evolutionary history has likely been influenced by diverse evolutionary trajectories.

To investigate the evolutionary conservation of the *PgALKBH* gene family, a comparative synteny analysis was performed between *ginseng* and *Arabidopsis* (Figure 4B). A total of 12 orthologous gene pairs involving nine *PgALKBH* genes were identified between the two species (Table S3). These included *PgALKBH1D-1*, *PgALKBH1D-2*, *PgALKBH1D-3*, *PgALKBH1D-4*, *PgALKBH8A*, *PgALKBH8B*, *PgALKBH9A-1*, *PgALKBH9A-2*, and *PgALKBH10*. Among these, *PgALKBH9A-1* and *PgALKBH9A-2* each exhibited syntenic relationships with two *Arabidopsis ALKBH* genes (*AtALKBH9B* and *AtALKBH9C*), while *PgALKBH10* showed synteny with *AtALKBH10A* and *AtALKBH10B*, indicating that these genes have retained conserved genomic relationships during evolution. In addition, members of the *PgALKBH1D* subfamily displayed conserved collinearity with *AtALKBH1B* and *AtALKBH1D*, whereas *PgALKBH8A* and *PgALKBH8B* were syntenic with *AtALKBH8B*. Overall, the conserved syntenic relationships between *ginseng* and *Arabidopsis* suggest that several *PgALKBH* genes have been evolutionarily conserved following species divergence.

An intra-specific collinearity analysis of the ginseng genome was conducted to understand the evolutionary expansion of the *PgALKBH* gene family, resulting in the identification of seven distinct pairs of syntenic *PgALKBH* genes. Most of these gene pairs are located on corresponding chromosomes within the subgenomes A and B, except for *PgALKBH2A* and *PgALKBH2B* (Figure 4C). The selection forces on duplicated *PgALKBH* genes were determined by analyzing their non-synonymous (Ka) and synonymous (Ks) substitution rates, along with the Ka/Ks ratio. The calculated Ka/Ks values for every gene pair were found to be lower than 1, falling within the range of 0.265 to 0.953 (Table S4). This outcome suggested that the evolution of the *PgALKBH* gene family has been mainly governed by purifying selection.

### 3.6. Identification of Putative Cis-Acting Regulatory Elements in PgALKBH Gene Promoters

**Figure 5:**
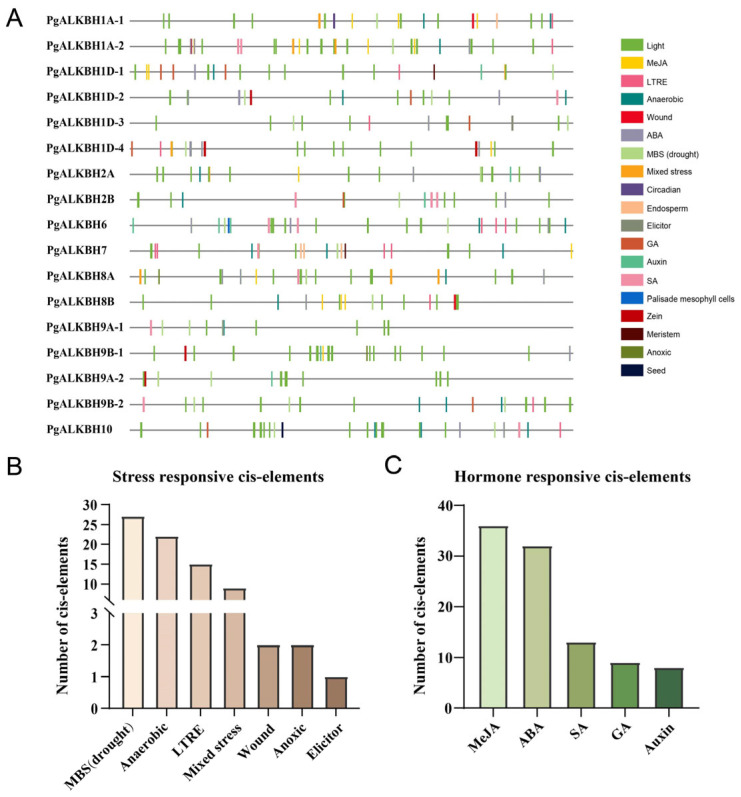
Analysis of predicted *cis*-acting regulatory motifs in the *PgALKBH* gene family. (**A**) Visualization of the layout for predicted *cis*-elements within the promoter sequences of each *PgALKBH* gene. In the diagram, the gray lines signify the upstream sequences. Specific *cis*-acting motifs are indicated by colored squares, with a corresponding legend provided on the right. (**B**) The abundance and types of predicted *cis*-elements related to various stress responses. (**C**) The abundance and categories of identified *cis*-elements associated with phytohormone signaling.

Understanding the *cis*-regulatory elements that control the transcriptional regulation of *PgALKBH* genes is essential for uncovering their biological roles in ginseng. An analysis of the *PgALKBH* genes revealed 386 *cis*-acting elements corresponding to nineteen distinct functional groups (Figure 5A). These were then classified into five principal types: those responsive to light, hormones, developmental cues, and stress, along with a category for other miscellaneous elements. A total of 192 light-responsive elements were identified, representing the largest category of regulatory elements and occurring in all *PgALKBH* gene promoters (Table S5). A considerable number of motifs involved in stress signaling were also found. More than 60% of these were drought-responsive MYB binding sites (MBS) and anaerobic induction elements, highlighting their importance in stress responses (Figure 5B). A number of hormone-responsive regulatory loci were found, including those that respond to methyl jasmonate (MeJA), ABA, gibberellin (GA), auxin, and salicylic acid (SA). The presence and arrangement of *cis*-acting elements in promoter regions suggest that MeJA is the dominant regulator of *PgALKBH* genes (Figure 5C). Moreover, the analysis also identified *cis*-acting elements that are involved in developmental processes and various other roles (Figure 5A). The abundance of light-, ABA-, MeJA-, and anaerobic-responsive elements in *PgALKBH* promoters suggested that their expression is regulated by light, phytohormones, and environmental stresses, which likely influence ginseng development.

### 3.7. PPI Network of PgALKBH Proteins

**Figure 6:**
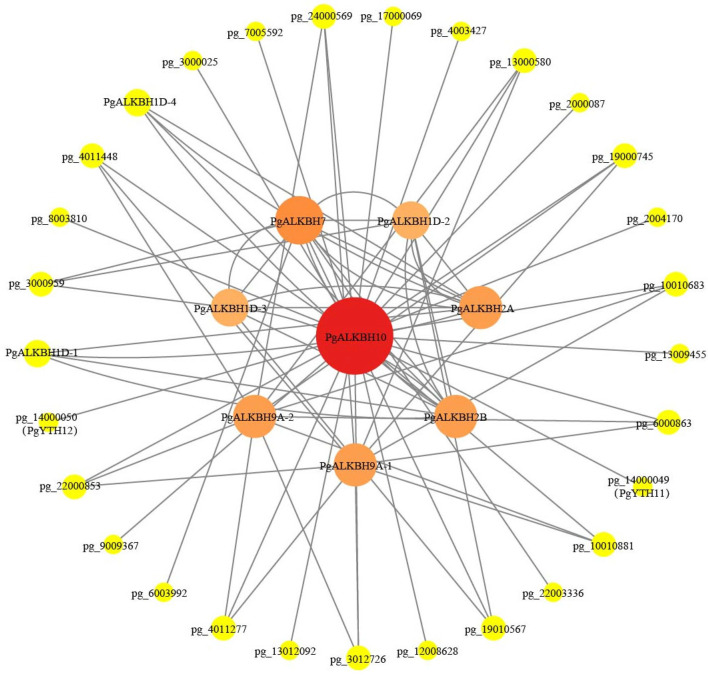
Analysis of PPI network of PgALKBH proteins. The darker the color, the higher the node degree.

To explore the potential functions of PgALKBH proteins, a PPI network was predicted based on *Arabidopsis* orthologs using TBtools and visualized with Cytoscape (Figure 6). PgALKBH10 was identified as the central hub with the highest connectivity, followed by PgALKBH7, PgALKBH2A, PgALKBH2B, PgALKBH9A-1/2, PgALKBH1D-2, and PgALKBH1D-3, whereas PgALKBH1D-1 and PgALKBH1D-4 were peripheral nodes with fewer interactions.

Functional annotation revealed that the interacting partners encompassed core components of the m^6^A regulatory system (Table S6). These included m^6^A writers, represented by N6-adenosine-methyltransferase MT-A70-like proteins pg_17000069 and pg_3000025, and m^6^A readers, represented by the two YTH-domain proteins PgYTH11 and PgYTH12 that were previously characterized by our group [40]. Together with the PgALKBH proteins themselves, which function as m^6^A erasers, the network thus encompassed all three core components of the m^6^A regulatory system. Several uncharacterized proteins were also present as potential novel interactors. The co-occurrence of m^6^A writers, readers, and erasers within a single PgALKBH-centered network suggests that PgALKBH proteins, particularly the hub protein PgALKBH10, may participate in m^6^A-mediated RNA regulation in ginseng through coordinated interactions with these functional partners.

### 3.8. Transcriptional Profiling of the Ginseng ALKBH Gene Family

**Figure 7:**
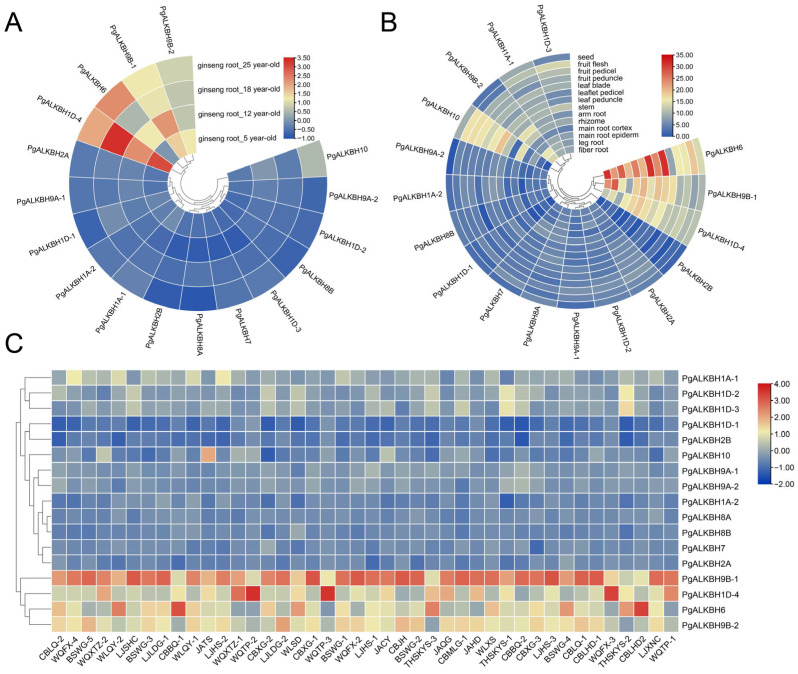
Expression patterns of *PgALKBHs*. (**A**) Transcriptional profiling of *PgALKBH* genes in ginseng roots across different growth stages (5, 12, 18, and 25 years). (**B**) Expression levels within fourteen distinct tissues from a four-year-old ginseng plant. These samples included the seed, fruit flesh, fruit pedicel, fruit peduncle, leaf blade, leaflet pedicel, leaf peduncle, stem, arm root, rhizome, main root cortex, main root epidermis, leg root, and fiber root. (**C**) Expression profiles of *PgALKBH* genes in roots from 42 different farm cultivars of 4-year-old ginseng.

To characterize the transcriptional profiles of the *PgALKBH* gene family in ginseng, we analyzed expression data derived from a diverse set of samples. This dataset included root tissues from four different growth years (5, 12, 18, and 25), a panel of 42 agricultural cultivars (S1–S42), and fourteen different plant tissues (Table S7). Among all *PgALKBH* genes, *PgALKBH1D-4* exhibited consistently high expression levels in roots across the four age groups, with TPM values of 70.35 (5-year-old), 26.12 (12-year-old), 57.27 (18-year-old), and 22.37 (25-year-old), which were generally higher than those of other family members, indicating that *PgALKBH1D-4* may be the predominantly expressed member of the *PgALKBH* gene family throughout root development. The expression level of *PgALKBH6* in 25-year-old roots was higher than in roots of the other ages, with TPM values of 24.65 (Figure 7A). Among the 17 *ALKBH* genes, *PgALKBH6* stood out for its markedly high expression levels across most of the fourteen tested ginseng tissues, with the notable exception of the leaf blade. This contrasts sharply with the remaining fourteen family members, which all demonstrated comparatively low transcriptional activity in these same tissues. This suggested that *PgALKBH6* may have distinct biological functions and regulatory roles in ginseng. The *PgALKBH9B-1* gene showed higher expression levels in leg root and fiber root, with TPM values of 27.50 and 23.30, respectively, than in other tissues (Figure 7B). Across 42 different *Panax ginseng* varieties, *PgALKBH9B-1*, *PgALKBH6*, *PgALKBH9B-2*, and *PgALKBH1D-4* exhibited higher expression levels than the other *PgALKBH* genes. In particular, *PgALKBH9B-1* displayed relatively high expression levels across all 42 varieties (Figure 7C).

### 3.9. Expression Analysis of PgALKBHs Under Abiotic Stress

**Figure 8:**
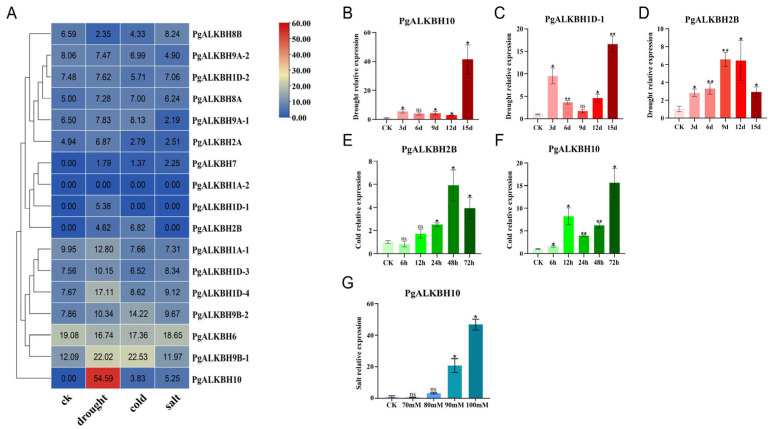
Expression profiling of *PgALKBH* genes under drought, cold and salt stress. (**A**) Heatmap showing the expression levels of *PgALKBH* family members under control, drought, cold, and salt stress conditions. (**B**–**D**) Expression patterns of *PgALKBH10*, *PgALKBH1D-1*, and *PgALKBH2B* in response to drought treatment over different time points (0, 3, 6, 9, 12, and 15 d). (**E**,**F**) Expression dynamics of *PgALKBH2B* and *PgALKBH10* under cold treatment at different time points (0, 6, 12, 24, 48, and 72 h). (**G**) Expression response of *PgALKBH10* to different salt concentrations (0, 70, 80, 90, and 100 mM). “*” indicates *p*  ≤  0.05; “**” indicates *p*  ≤  0.01; “ns” indicates not statistically significant.

To characterize the transcriptional profiles of the *PgALKBH* gene family under abiotic stress in ginseng, we analyzed RNA-Seq datasets from the Ginseng Genome Data Resource. The majority of *PgALKBH* genes showed no significant changes in expression following stress treatment. *PgALKBH10* showed increased expression across the three different stress conditions, particularly under drought stress (Figure 8A).

Based on differential expression analysis of the transcriptome (log_2_FoldChange > 1 and adjusted *p* < 0.01), candidate genes with prominent expression alterations were identified from the *PgALKBH* family. Subsequent qRT-PCR validation confirmed that these *PgALKBH* members exhibited distinct, condition-specific transcriptional responses to drought, cold, and salt stresses. *PgALKBH10*, *PgALKBH1D-1*, and *PgALKBH2B* exhibited significantly higher expression levels than the control at nearly all time points under drought treatment (Figure 8B-D). *PgALKBH2B* showed significant upregulation at 24, 48, and 72 h under cold stress, whereas *PgALKBH10* maintained significantly higher expression levels than the control throughout the entire time course (Figure 8E,F). *PgALKBH10* displayed a typical dose-dependent response, with no significant alterations in expression at low salt levels (70–80 mM). The expression of *PgALKBH10* increased with rising salt concentrations, particularly at 90 mM and 100 mM (Figure 8G). Functional divergence was observed among *PgALKBH* family members in ginseng. Notably, *PgALKBH10* was up-regulated under drought, low-temperature, and salt stress. The most pronounced increase in expression was observed during the late stage of drought and under high-salt treatment, suggesting that *PgALKBH10* may play a pivotal role in abiotic stress responses of ginseng.

Given the limited understanding of stress-related genes in ginseng, Pearson correlation analysis was performed to explore the potential associations between *PgALKBH* genes and annotated stress-responsive genes under drought, cold, and salt stress conditions (Table S8). The results showed that *PgALKBH1D-1*, *PgALKBH2B*, and *PgALKBH10* exhibited strong correlations (|*r*| > 0.95) with several stress-related genes (e.g., pg_9010504, pg_15003129, and pg_5002129), suggesting their involvement in multiple stress adaptation processes.

## 4. Discussion

A wide variety of biological processes are profoundly influenced by the m^6^A modification, which serves a pivotal function in governing the stability and translational efficiency of mRNA transcripts [41]. In mammals, the identification of m^6^A demethylases, primarily ALKBH5 and fat mass and obesity-associated protein (FTO), revealed that m^6^A modification is reversible [42,43]. This discovery has significantly accelerated research on the functional roles of m^6^A in various organisms [44]. More thorough and precise investigations of plant gene families have been made possible by the recent surge in available reference genomes. This wealth of genomic data is a direct result of the swift progress made in whole-genome sequencing technologies. Although no homolog of the FTO gene has been found in plants, the widespread presence of the *ALKBH* gene family across multiple plant species indicates a conserved mechanism for m^6^A demethylation in the plant kingdom. Previous studies have revealed variable numbers of *ALKBH* genes in different plants, such as 13 in *Arabidopsis* [45], 22 in soybean [46], 8 in oriental melon [47], and up to 30 in wheat [48], reflecting possible species-specific expansions or contractions related to their evolutionary histories and functional demands. To date, there has been no documentation of the *ALKBH* gene family in ginseng.

The present investigation led to the discovery and characterization of 17 distinct genes belonging to the *PgALKBH* family in *Panax ginseng*. The newly available T2T reference genome of ginseng was crucial for this analysis, as it provided a more complete and contiguous genomic framework than earlier draft assemblies [25]. This advantage is especially important in ginseng, a complex allotetraploid species, because the T2T genome enabled more reliable gene identification, more accurate chromosomal localization, and better resolution of duplicated and syntenic regions. As a result, our analysis provides a more comprehensive and confident overview of the *PgALKBH* gene family. The chromosomal distribution of *PgALKBH* genes in ginseng reveals an approximately equal presence on its two subgenomes, A and B, consistent with its allotetraploid nature [25]. The presence of genes on corresponding chromosomes across both A and B subgenomes further supports the idea of conserved genomic segments retained through ginseng’s complex evolutionary history, shaped by distinct evolutionary trajectories in each subgenome. The *ALKBH* genes were previously reported to be divided into seven subfamilies, with members generally grouped according to their homology to *Arabidopsis* ALKBH proteins [45]. Similar to *Arabidopsis*, most species have more members in the *ALKBH1*, *ALKBH9*, and *ALKBH10* subfamilies. However, the *ALKBH10* subfamily gene is absent in tomato, and there is only one *ALKBH10* gene in sweet orange. Other *ALKBH* subfamilies mostly contain 1–2 genes per subfamily across species, except that Populus has 4 genes in the *ALKBH8* subfamily and lacks *ALKBH7* subfamily genes [49]; pigeon pea lacks genes in the *ALKBH6* and *ALKBH7* subfamilies [50]. Wheat has 3 genes each in the *ALKBH2*, *ALKBH6*, *ALKBH7*, and *ALKBH8* subfamilies [48], highlighting the impact of polyploidy on *ALKBH* gene family expansion. Only one gene (*PgALKBH10*) belonging to the *ALKBH10* subfamily was identified in this study, suggesting lineage-specific gene loss or divergence affecting potential regulatory mechanisms mediated by the *ALKBH10* subfamily. This may have significant implications for m^6^A-mediated regulatory mechanisms, potentially affecting specific pathways reliant on *ALKBH10*-mediated demethylation.

The expression patterns of the *ALKBH* gene family across different plant species indicate a strong conservation of tissue-specific roles, highlighting their potential importance in plant development and function. Huang et al. reported that nearly all *CsALKBH* genes in sweet orange show high expression in callus, while *CsALKBH9A* exhibits relatively low expression in roots and leaves [51]. This is similar to the low expression of *PgALKBH9A* in roots and leaves observed in our study. Zhao et al. found that members of the *ALKBH1*, *ALKBH6*, and *ALKBH9* subfamilies are highly expressed in Populus leaves [49]. This corresponds with our findings that *PgALKBH1D-4*, *PgALKBH6* and *PgALKBH9B-1* also show strong expression in leaves, suggesting these *ALKBH* members may have conserved roles in regulating leaf function. In cotton, *GhALKBH1D* was reported to be highly expressed in roots [18], consistent with the high-expression pattern of *PgALKBH1D-4* in our study. This result implies a comparable function for the gene in the growth, development, or overall biological activity of roots across various species. In potato and wheat, *StALKBH9B*, *StALKBH10*, *TaALKBH9B* and *TaALKBH10* are significantly up-regulated in stem and leaf tissues [48,52]. Likewise, *PgALKBH9B-1* and *PgALKBH10* in our research also show high expression in stems and leaves, further supporting the notion that *ALKBH9B-1* and *ALKBH10* genes may have conserved functions in the aerial parts of plants. The conserved expression trends highlight evolutionary pressure to maintain *ALKBH*-mediated m^6^A regulation in specific tissues, while observed expression differences among species may enable specialized adaptations.

Members of the *ALKBH* family have emerged as important regulators of plant growth, development, and environmental adaptation through their roles in RNA m^6^A demethylation. Increasing evidence indicates that ALKBH-mediated epitranscriptomic regulation participates in abiotic stress signaling pathways by modulating the expression of stress-responsive genes. In *Arabidopsis*, the demethylase *ALKBH9C* confers salt tolerance by regulating the transcription levels of the positive effectors SOS1, SAD1, PIP1D and the negative factor PATL1 [53]. In tomato, knocking out *SlALKBH9B* increased flower drop, whereas overexpressing *SlALKBH9B* delayed the onset of flower drop. m^6^A modification inhibits drought-induced flower drop by regulating the ethylene synthesis pathway [54]. A study on sugar beet found that salt stress induced diverse expression of *BvALKBH genes* in its leaves, with *BvALKBH10B* being significantly upregulated while *BvALKBH9B* was strongly repressed [55]. Research on potato revealed that several *StALKBH* family members, including *StALKBH9B* and *StALKBH10B*, were significantly upregulated under salt stress but generally downregulated during cold treatment, indicating that the same genes may be differentially regulated under varying stress conditions [52]. Similarly, a study on soybean showed that different genes within the *GmALKBH10* subfamily responded to cold, alkaline, and drought stresses with varying intensities and timing. Moreover, the expression peaks of different *GmALKBH10* genes after cold treatment occurred at different time points, suggesting their distinct roles during early, middle, and late phases of stress responses [46].

In the present study, transcriptome analysis and qRT-PCR validation revealed that several *PgALKBH* genes responded significantly to drought, cold, and salt stresses, indicating their potential involvement in abiotic stress adaptation in ginseng. The differential expression patterns observed among *PgALKBH* genes indicate substantial functional diversification within the ginseng *ALKBH* family. Such divergence may enable ginseng to fine-tune epitranscriptomic regulation under varying environmental conditions, thereby improving stress adaptability. Among all examined genes, *PgALKBH10* emerged as the most promising candidate involved in abiotic stress responses. In addition, the predicted PPI network identified PgALKBH10 as the central hub of the PgALKBH proteins, while Pearson correlation analysis revealed strong positive correlations between *PgALKBH10* and multiple stress-responsive genes. Given the established functions of ALKBH proteins as m^6^A demethylases, *PgALKBH10* may regulate the stability, splicing, transport, or translation of stress-responsive transcripts through dynamic RNA methylation changes.

Nevertheless, several limitations of the present study should be acknowledged. Although our study identified *PgALKBH10* as a promising candidate involved in abiotic stress responses, these findings are primarily based on expression profiling and bioinformatic analyses and therefore do not provide direct evidence of its biological function. In addition, stress-specific treatment conditions were selected according to commonly used experimental protocols for each type of abiotic stress. Consequently, the drought, cold, and salt stress experiments differed in treatment duration, sampling time points, and salt concentrations, which may limit direct comparisons among different stress responses. Taken together, these limitations indicate that the precise molecular mechanisms underlying the function of *PgALKBH10* remain to be elucidated. Future studies involving gene overexpression, knockout or knockdown approaches, together with transcriptome-wide m^6^A profiling and identification of downstream target genes, will be necessary to clarify the molecular mechanisms and regulatory pathways mediated by *PgALKBH10*. Such investigations will advance our understanding of m^6^A-mediated epitranscriptomic regulation in ginseng stress responses and may provide valuable genetic resources for improving stress tolerance in cultivated ginseng varieties.

## 5. Conclusions

In this study, we performed the first comprehensive genome-wide characterization of the *ALKBH* gene family in ginseng using the T2T reference genome. A total of 17 *PgALKBH* genes were identified and systematically characterized through analyses of phylogenetic relationships, gene structures, conserved motifs, chromosomal distribution, syntenic relationships, *cis*-acting regulatory elements, 3D protein structures, PPI networks, and expression profiles. The results showed that the *PgALKBH* gene family is highly conserved during evolution while exhibiting evidence of functional diversification. *PgALKBH10* was identified as a promising candidate associated with abiotic stress responses. However, its biological function remains to be experimentally validated. Overall, this study provides a comprehensive resource for understanding the evolution and potential functions of the *PgALKBH* gene family in ginseng and establishes a solid foundation for future functional studies on m^6^A-mediated epigenetic regulation and the molecular breeding of stress-tolerant ginseng cultivars.

## Figures and Tables

**Table 1:** Protein characteristics of predicted *PgALKBH* genes in ginseng.

Gene Name	Gene ID	Number of Amino Acid	Molecular Weight (MW)	Isoelectric Point (pI)	Location	Subcellular Localization
PgALKBH1A-1	pg_1000211.t01	360	40,670.4	5.9	Chr01:1796529-1800141(−)	Nucleus
PgALKBH1A-2	pg_9011448.t01	330	37,376.71	6.04	Chr09:146692600-146695997(−)	Nucleus
PgALKBH1D-1	pg_3013481.t01	300	33,350.11	7.63	Chr03:175959207-175962520(+)	Nucleus
PgALKBH1D-2	pg_4011596.t01	362	40,924.44	8.51	Chr04:170672519-170675042(−)	Cytoplasm
PgALKBH1D-3	pg_10011064.t01	387	43,619.81	9.47	Chr10:144323551-144326425(−)	Plastid
PgALKBH1D-4	pg_19000070.t02	408	45,197.03	8.66	Chr19:829595-834230(−)	Nucleus
PgALKBH2A	pg_23000155.t01	244	27,972.02	8.97	Chr23:1456106-1458537(−)	Nucleus
PgALKBH2B	pg_24007950.t01	241	27,920.07	9.51	Chr24:104287641-104291106(+)	Nucleus
PgALKBH6	pg_14000020.t01	258	29,141.97	6.3	Chr14:266182-272568(+)	Nucleus
PgALKBH7	pg_20002311.t02	217	24,436.63	7.1	Chr20:37126701-37128330(−)	Nucleus
PgALKBH8A	pg_4000687.t01	265	30,477.21	4.83	Chr04:6798400-6803653(−)	Nucleus
PgALKBH8B	pg_10000671.t01	267	30,693.46	4.63	Chr10:5711525-5716627(−)	Nucleus
PgALKBH9A-1	pg_6001170.t01	508	57,094.53	6.66	Chr06:11396976-11401426(−)	Nucleus
PgALKBH9A-2	pg_22001132.t01	508	57,116.48	6.11	Chr22:9624431-9628852(−)	Nucleus
PgALKBH9B-1	pg_8011724.t01	472	52,788.3	8.67	Chr08:158383631-158389060(+)	Cytoplasm
PgALKBH9B-2	pg_15008195.t01	560	62,582.53	8.94	Chr15:111006245-111013244(+)	Cytoplasm
PgALKBH10	pg_5000707.t01	612	67,908.19	6.7	Chr05:6197201-6203803(−)	Nucleus

## Data Availability

The original contributions of this study are contained within the article/Supplementary Material.

## Supplementary Materials

The following supporting information can be downloaded at: https://www.mdpi.com/article/10.3390/genes17070793/s1, Table S1: Plant materials used for this study; Table S2: Primers used for qRT-PCR; Table S3: Gene pairs between Arabidopsis and Ginseng; Table S4: The KaKs ratios and estimated divergence times for duplicate pairs of *PgALKBHs*; Table S5: *Cis*-acting elements of *PgALKBHs*; Table S6: PPIs and credibility scores for *PgALKBH* family members in ginseng; Table S7: The TPM values of 17 *PgALKBH* genes in 42 ginseng varieties; Table S8: Pearson correlation analysis of *PgALKBH* genes under drought (|*r*| > 0.95).
